# Germany-wide evaluation of residency in neurological intensive care medicine

**DOI:** 10.1186/s12909-022-03441-4

**Published:** 2022-05-12

**Authors:** Ann-Kathrin Ernst, Michaela Zupanic, Gisa Ellrichmann, Anne-Sophie Biesalski

**Affiliations:** 1 Faculty of Medicine, Ruhr-University Bochum, Universitätsstrasse 150, 44801 Bochum, Germany; 2 Ruhr-University Bochum, Gudrunstrasse 56, 44791 Bochum, Germany; 3 Faculty of Medicine, University Witten/Herdecke, Alfred-Herrhausen-Strasse 45, 58455 Witten, Germany; 4 Department of Neurology, Klinikum Dortmund, Beurhausstrasse 40, 44137 Dortmund, Germany; 5 Department of Neurology, St. Josef Hospital, Gudrunstrasse 56, 44791 Bochum, Germany

**Keywords:** Neurology, Intensive care medicine, Neurointensive, Residency, Teaching, Education

## Abstract

**Background:**

Neurointensive medicine is an important subspecialization of neurology. Its growing importance can be attributed to factors such as demographic change and the establishment of new therapeutic options. Part of the neurological residency in Germany is a six-month rotation on an intensive care unit (ICU), which has not yet been evaluated nationwide. The aim of this study was to evaluate kind and feasibility of neurointensive care training in Germany and to discover particularly successful training concepts.

**Methods:**

In a preliminary study, ten residents and ten instructors were interviewed. Using content analysis, two questionnaires were created, which contained questions about specific teaching methods as well as individual satisfaction. The questionnaires were sent to 187 neurological clinics in Germany, and residents and instructors were asked to participate in the study. The data analysis was performed using SPSS and content analysis for the free-text data.

**Results:**

Seventy of the 187 clinics contacted did not offer ICU-rotation. At 59,8% (*n* = 70) of the remaining hospitals, a total of 154 participants (84 residents, 70 educators) could be recruited. General satisfaction with the neurointensive medical training is high in both groups (residents: 3.34 ± 0.54; instructors: 3.79 ± 0.41, evaluated on the basis of a Likert scale from 1 = “not satisfied” to 5 = "fully satisfied"). Specific teaching methods (e.g. simulation trainings, feedback sessions) are perceived as very useful by residents, but rarely take place. Instructors are interested in educational opportunities such as didactic courses.

**Conclusion:**

This study provides an overview of the ICU-rotation as part of the five-year neurological residency. Neurointensive care rotations usually take place at maximum care hospitals and last at least seven months. Despite frequent time and personnel restrictions, motivation of trainers and residents is high. Nevertheless, teaching methods as simulation training and educational opportunities for instructors must be expanded.

## Background

Neurological intensive care medicine (NICM) is an important specialization in neurology. The wide number of options, e.g. in stroke therapy, as well as the rising age of the population with increasing frequency of neurological diseases, lead to a growing number of intensive care treatments [1]. In addition, COVID-19 patients requiring intensive care medicine suffer from different neurological complications [2] which also increases the importance of NICM.

At the same time, the number of neurologists in Germany is growing, most recently in 2019 by 5.7% (compared to the previous year) [3]. Part of the german specialist training in neurology is an obligatory six-month basic training in intensive care medicine. Longer periods can also be credited. A two-year fellowship in neurointensive medicine can be granted after passing the specialist examination in order to get the title of intensive care medicine specialist [4, 5].

In fact, however, the six-month residency cannot always be guaranteed to assisting physicians. Busse et. al. [6], who evaluated intensive care units (ICUs) in 2018, provided initial data: Only 12.2% of the hospitals with certified stroke units have an independent neurological intensive care unit (NICU). In many cases, stroke unit rotations are accepted as a substitute for working in an ICU [6].

Currently, no data are available on the duration and quality of neurology intensive care training in Germany.

Throughout Europe, the guidelines differ considerably: a survey by Kleineberg et al. [7] showed significant international diversity regarding the duration of intensive care training while in residency. In about 15 countries carrying out intensive care rotation, it lasts between one and six months. Although the study did not collect data on the quality of intensive care training in particular, it called for a more uniform concept as it was defined by the European Academy of Neurology (EAN) [7].

In the USA, neurological residency does not necessarily include intensive care rotation. If it does, it often lasts a maximum of four weeks [8]. Similar to Germany, the US-residency can be followed by a specialized, two-year fellowship. However, an evaluation of the fellowship by the Physician Certification and Fellowship Training Committee of the Neurocritical Care Society in 2015 showed a great heterogeneity in the type of neurointensive medical training and a lack of consistency in requirements for fellow procedural competence [9]. The aim of our study was to evaluate kind and feasibility of neurointensive care training in Germany and to discover particularly successful training concepts.

## Methods

In order to develop a questionnaire, qualitative interviews were conducted with neurological residents (*n* = 10) and instructors (*n* = 10) in ICUs as part of a preliminary study (Note: In the following, the term "instructor" refers to the specialist who instructs the residents on the ICU). The interview guide was based on experiences with varying teaching methods and perceived individual development during the neurological medical training. After deductive content analysis of the interviews, two questionnaires were designed—one for the instructors and one for the residents. In addition to questions on specific teaching methods, they contained Likert scales for assessing individual satisfaction and two free text questions. The sum value of the items that were determined to both groups served as level for satisfaction of the neurological medical training in both groups. The internal consistency of those items was examined and showed a good Cronbach's alpha > 0.780 for both groups. Validation of the questionnaire was carried out in the present study. Inclusion criteria for the residents were at least two months of regularly working in the ICU as part of their neurological residency or finishing ICU training no longer than four months ago.

Participation in the preliminary or main study in all cases was voluntary and could be stopped at any time without giving reasons. Both parts of the study received a positive ethics vote from the Ruhr-University of Bochum (Registration numbers: 18–6535-AMG, 18–6693-BR).

For recruitment we used a list of ICUs treating neurological patients in Germany published by the German Society for Neurointensive and Emergency Medicine (DGNI) [10]. After removing clinics with exclusively neurosurgical ICUs, 187 hospitals in Germany remained.

Initially, chief physicians were asked for assistance with the study. If this failed, the hospitals were contacted by phone or we used personal acquaintances in consultation the German Society of Neurology (DGN), the DGNI as well as the Network “Junge Neurologen”. Physicians who agreed to participate could either complete the questionnaire using the online platform or be interviewed by telephone. The participation period was set between September 13, 2019 and April 30, 2020.

The analysis of the data was carried out descriptively as well as inductively (comparison of groups with analyses of variance, ANOVA) using SPSS (version 26). The two free text questions were tagged with codes and analyzed by summarizing content analysis according to Mayring (2014) [11].

## Results

Seventy of the 187 hospitals contacted did not offer a rotation to an ICU. Based on the remaining 117 clinics, a total of 154 participants (84 residents, 70 instructors) were recruited at 70 locations (59,8%). In some cases, several representatives of a group answered the questionnaire (Table 1).

**Table 1:** Properties of the participants

		Residents n (%)^a^	Instructors n (%)^a^	
**Gender**	*male*	41 (48.8)		53 (75.7)
	*female*	43 (51.2)		15 (21.4)
	*other*	-		1 (1.4)
**Age**	*under 31*	40 (47.6)		1 (1.4)
	*31–38*	43 (51.2)		10 (14.3)
	*39–47*	1 (1.2)		34 (48.6)
	*over 47*	-		25 (35.7)
**Years of Professional**	*under 2*	14 (16.7)	*under 10*	5 (7.1)
	*2–5*	49 (58.3)	*10–15*	22 (31.4)
	*over 5*	21 (25.0)	*over 15*	43 (61.4)

^a^Percentages relate to the group of residents (*n* = 84) respective instructors (*n* = 70)

The majority of the surveyed residents (48.8% male) were in the advanced stage of their education. 47.6% (*n* = 40) were in the third/fourth year of their residency and 32.1% (*n* = 12) were at least in the fifth year of residency. Of the instructors, 85.7% (*n* = 60) were specialists in neurology, and 81.4% (*n* = 57) had an additional specialty in critical care medicine in addition to their initial specialty.

The majority of respondents in both groups work in maximum care facilities, mostly publicly funded, at university hospitals or at an academic teaching hospital (Table 2). 84.3% (*n* = 59) of the surveyed specialists could offer fellowships in "intensive care medicine" at their hospital.

**Table 2:** Features of the clinics

		Residents n (%)^a^	Instructors n (%)^a^
**Ownership**	*nonprofit*	4 (4.8)	4 (5.7)
	*private*	7 (8.3)	10 (14.3)
	*public*	73 (86.9)	56 (80.0)
**Supply level**	*maximum treatment*	75 (89.3)	53 (75.7)
	*specialized care*	8 (9.5)	15 (21.4)
	*basic/regulated supply*	1 (1.2)	2 (2.9)
**Number of beds in the neurological clinic**	*up to 30*	11 (13.1)	3 (4.3)
	*31–60*	25 (29.8)	12 (17.1)
	*from 61*	48 (57.1)	50 (71.4)
**Number of beds in the ICU**	*up to 10*	24 (28.6)	24 (34.3)
	*11–15*	36 (42.9)	18 (25.7)
	*from 15*	23 (27.4)	28 (40.0)
**Type of ICU**	*neurological*	46 (54.8)	33 (47.1)
	*neurological-neurosurgical*	9 (10.7)	8 (11.4)
	*interdisciplinary-operative*	5 (6.0)	2 (2.9)
	*interdisciplinary-conservative*	21 (25.0)	20 (28.6)
	*other*	2 (2.4)	7 (10.0)
**Time spent on documentation**	< *25% of working time*	13 (15.5)	-
	*25–50% of working time*	59 (70.2)	-
	> *50% of working time*	11 (13.1)	-

^a^Percentages relate to the group of residents (*n* = 84) respective instructors (*n* = 70)

### Organization in the ICU

45.8% of the residents were responsible for four to eight patients in the ICU, 35.7% (*n* = 30) treated more than eight patients. 40.5% (*n* = 34) of the residents additionally took care of patients on the intermediate care ward. 85.7% (*n* = 72) of the residents had to work in night and weekend shifts.

### Rotation

54.3% (*n* = 33) of the instructors stated that the time for rotation to the ICU is not determined when residents start working in the neurological department. More than half of the residents (*n* = 37, 52.9%) have the option of doing their ICU rotation part-time.

For 67 residents (80.7%) an obligatory contact person during ICU time was present, 46 (55,4%) had a regular debriefing. Further information on ICU teaching (*n* = 83, 1 = missing) is given in Table 3.

**Table 3:** Information on rotation and gauge

		Residents n (%)^a^
**Number of neurological assistants rotating simultaneously to the ICU**	*1–3*	33 (39.8)
	*4–6*	21 (25.3)
	*7–9*	18 (21.6)
	*10 or more*	10 (12.0)
**Weeks of training before first (night/weekend) duty**	*up to 2*	5 (6.0)
	> *2–4*	20 (24.1)
	> *4–6*	12 (14.4)
	> *6*	34 (40.9)
**Duration of the rotation**	*less that 4 months*	1 (1.2)
	*4–6 months*	18 (21.7)
	*7–10 months*	24 (28.9)
	> *10 months*	41 (49.4)
**Time of the (first) rotation**	*1st/2nd year of residency*	32 (38.6)
	*3rd/4th year of residency*	41 (49.4)
	*After the 4th year of residency*	11 (13.2)
**Pure training period at the start of the rotation**	< *1 week*	30 (35.1)
	*1–2 weeks*	18 (21.7)
	*3–4 weeks*	23 (27.7)
	> *4 weeks*	12 (14.4)

^a^Percentages relate to the group of residents (*n* = 83, 1 = missing)

### Training

57.1% (*n* = 48) of the residents and 82.9% (*n* = 58) of the instructors had a written introductory concept for the ICU. More than half of the residents (58.3%, *n* = 49) were mainly introduced by other residents (from neurology or another specialty) or other non-medical professional groups such as nursing staff (11.9%). 58.3% (*n* = 49) of the participating residents had completed an internship in anesthesiology as part of their introductory training.

### Continuing education and teaching

62.9% (*n* = 44) of the instructors had no opportunity to plan or conduct residency training during their working hours. 20.0% (*n* = 14) were rarely and 11.4% (*n* = 8) irregularly released from work, 5.7% (*n* = 4) regularly or daily. Most of the resident’s self-study must be done outside working hours. Only three of the residents had fixed times for self-study during their working hours. The average time for self-study was 3.22 h per week. Nearly one-third (30.0%, *n* = 21) of the instructors had received training (e.g. didactics course) in teaching or further education. However, the majority (65.7%, *n* = 46) had not yet taken part in educational training.

39.3% (*n* = 33) of the residents and 74.3% (*n* = 52) of the instructors could perform simulation training sessions in ICU. 81.0% (*n* = 68) of the residents and 98.6% (*n* = 69) of the instructors had standard operating procedures (SOPs) for their ICUs.

About one third (31.4%, *n* = 22) of the instructors administered a knowledge test before the residents worked alone for the first time. 60.0% (*n* = 42) of the instructors and 82.1% (*n* = 69) of the residents declared that no test was performed before the first shift.

### Documentation of mistakes and evaluation

82.9% (*n* = 58) of the instructors and 45.2% (*n* = 38) of the residents could document mistakes in a special system, but only three (7.9%) of the residents had already used it. 23.8% (*n* = 20) of the residents did not even know whether such a system existed for the ICU. According to 68.6% (*n* = 48) of the instructors, the quality of continuing education was assessed internally. Once again, the residental information differed from that of the instructors: 46.4% (*n* = 39) of the residents denied evaluation of teaching in their hospital, or could not provide any information on this (41.7% (*n* = 35) "do not know"). In only 11.9% (*n* = 10) of the residents the quality of continuing education in ICUs was regularly assessed.

### Individual satisfaction

Table 4 shows information on the individual satisfaction of both groups, residents (*n* = 83, 1 = missing) and instructors (*n* = 69, 1 = missing). The majority of the interviewees of both groups responded to be "satisfied with the training in the ICU".

**Table 4:** Individual satisfaction

	Residents (*n* = 83)mean ± standard deviation		Instructors (*n* = 69)mean ± standard deviation		F-value (*p*-value)
I enjoy working in the ICU	4.22	0.90	4.62	0.81	8.12 (.005)
I enjoy working as a clinical educator	-	-	4.75	0.50	-
I am satisfied with the training in the ICU	3.67	0.95	3.58	1.06	n. s
I can easily combine work in the ICU with my private life	3.05	1.13	-	-	-
My supervisors show me appreciation	4.01	0.86	4.18	0.88	n. s
There is a good working atmosphere in the ICU	3.89	1.00	4.13	0.81	n. s
I feel ethically burdened by the work in the ICU	3.20	1.30	3.60	1.12	4.14 (.044)
I feel psychologically burdened by the work in the ICU	3.25	1.27	3.66	1.03	4.44 (.037)
I feel physically stressed by the work in the ICU	2.88	1.15	3.63	1.10	16.56 (.000)
I feel sufficiently trained for the activities in the ICU. / After the training period, residents are sufficiently trained for work in the ICU	3.33	1.11	3.58	0.99	n. s
I feel safe during activities such as the placement of central venous catheters, intubation, etc. / Activities such as the placement of central venous catheters, intubation etc. are safely mastered by the resident	3.43	1.17	3.88	0.98	6.46 (.012)
It is always possible for me/the resident to ask a specialist for advice and/or help	3.99	1.06	4.81	0.47	n. s
My supervisors support my work as an educator	-	-	3.88	0.91	-
I feel sufficiently trained in making ethically difficult decisions	3.11	0.90	-	-	-
I am afraid of doing something wrong at work in the ICU	2.60	1.15	-	-	-
When working with critically ill patients, I am afraid to make mistakes	2.51	1.08	-	-	-
I have already made mistakes at work that could have been avoided by better training	3.45	1.00	-	-	-
I would like to have a permanent contact person who provides me with professional support during the rotation in the ICU	3.98	1.17	-	-	-
I dare to speak openly about grievances in my ICU	3.87	0.78	-	-	-
I / Residents have the opportunity to actively participate in the continuing education in the ICU	3.54	0.98	3.78	0.97	n. s
Formal feedback is regularly given during rotation in the ICU	2.30	1.23	3.62	0.96	52.35 (.000)
I would like to have regular feedback. / I regularly give the residents a structured feedback for their work	1.85	0.69	2.33	0.92	13.44 (.000)
Length of stay or duration of ventilation in my ICU are influenced by economic factors	3.40	1.18	-	-	-
I consider an examination of the intensive care knowledge (e.g. before the first service) to be useful to ensure the quality of patient care	3.70	1.12	3.72	1.15	n. s
If mistakes are made at work, these are discussed in the team and possibilities for improvement are sought together	3.30	1.02	4.28	0.78	42.15 (.000)
Before my rotation to the ICU, I was afraid of the confrontation with critically ill patients	2.38	1.24	-	-	-
I would like to have a better education as a clinical educator	-	-	2.85	1.25	-

Some statements were evaluated only by the representatives of one group

The sum value of the items that were determined to both groups served as level for satisfaction of the neurological medical training (residents: 3.34 ± 0.54; instructors: 3.79 ± 0.41)

The internal consistency of those items was examined and showed a good Cronbach's alpha > 0.780 for both groups

The statements were evaluated on the basis of a Likert scale (1 = "Do not agree at all", 2 = "Rather not agree", 3 = "Neutral", 4 = "Rather agree", 5 = "Fully agree")

Results of ANOVA are given with F- and *p*-value (n. s. = non significant)

The evaluation of the two free text questions about well-functioning training concepts was carried out through a content analysis (Tables 5 and 6). A total of 100 participants (*n* = 55 residents; *n* = 45 instructors) provided information about their own model. 33 participants had no structured further training concept for ICU-rotation or – if one exists—it cannot be adhered to because of less staff or time. A written training manual or an underlying "checklist" existed in 21 cases. Intubation training in anesthesiology was reported 15 times. It lasted between one and eight weeks or, in two cases, took place daily in the morning before work. Nine participants had the opportunity to take part in internal or external further training courses as part of ICU further training. Six of the participants said that teaching in ICU is like "doing the tasks on your own and being corrected later". A structured examination of skills and abilities by an experienced specialist was reported three times. Other kinds of teaching were: internal mentoring system (*n* = 1, feedback interviews (*n* = 3), case seminars (*n* = 2) or simulation training (*n* = 2).

**Table 5:** Existing well-functioning training concepts on ICUs

**Does your clinic have a well-functioning concept of intensive medical training? Please describe it briefly**	**Number n (%)**^a^
*No structured further training concept or not feasible due to lack of time or personnel (e.g. "There is no clearly standardized concept available")*	33 (32.7)
*Written training manual or "checklist" available (e.g. "Self-created document of the educator to be checked off by the resident")*	21 (20.8)
Intubation training in in-house anesthesiology (e.g. "…daily airway management in anesthesiology" or "anesthesiology hospitation before starting")	15 (14.9)
Clinic-internal or external advanced and further training (e.g. "Integration in advanced training courses in intensive care medicine")	9 (8.9)
"Trial and error" concept (e.g. "…the concept is to just do it and be corrected")	6 (5.9)
Structured examination of knowledge by an experienced specialist or senior physician (e.g. "…in discussions it is checked where there are deficiencies and the training is adjusted")	3 (2.9)
Simulation training (e.g. "Three afternoons per year in the simulation center (…), simulation of neurological clinical pictures, including anesthesiological hands-on-teaching")	2 (2.9)
Regular feedback sessions (3 mentions), Case seminars (2 mentions), Internal hospital mentoring system (1 mention)	(< 3)

^a^In some cases several mentions within one free text; Percentages relate to all responses (*n* = 101)

**Table 6:** Proposals for the improvement of intensive care training

**How can further education at the ICU be promoted?**	**Number n (%)**^a^
More time/personnel/resources for continuing education (both self-study and teaching through continuing education) as well as restructuring of the personnel situation (e.g. "fewer patients for working specialists to ensure better continuing education" or: "no parallel responsibilities")	48 (44.0)
Establishment of internal and external training and further education, support of e.g. congress participation (e.g. "regular internal further education" or: "Resident doctors should design their own further education")	34 (31.2)
Improved/structured/longer familiarization training, creation of written templates/SOPs/logbooks (e.g. "fixed, several months' familiarization training)	33 (30.3)
Rotation to other departments, intubation training in anaesthesia (e.g. "Rotation to the anaesthesiological clinic to learn how to intubate")	26 (23.9)
Fixed contact persons, specialist physician standard (e.g. "fixed specialist physician contact persons (for residents)" or: "…continuous specialist physician presence at the ICU")	21 (19.3)
More interdisciplinarity, cooperation with other departments, common visits (e.g. "regular interdisciplinary visits and case discussions")	16 (14.7)
Regular feedback meetings, structured supervision, debriefing of difficult / stressful situations (e.g. "regular, structured feedback" or: "consultation after stressful situations (preferably moderated)")	14 (1.8)
Regular simulation training, training on equipment (e.g. "regular training of practical skills…" or: "… (simulation) training of critical situations")	13 (11.9)
Structured knowledge check, e.g. before the official capacity (e.g. "obligatory knowledge check after the initial training…")	9 (8.3)
Single entries: Establishment of a purely neurological ITS (3 denominations), connection with German Society for Neurology (DGN), German Society for Neurological Intensive Care Medicine (DGNI), German Medical Association or similar (2 mentions)	(< 3)

^a^In some cases several mentions within one free text; percentages relate to all responses (*n* = 109)

## Discussion

The results of our survey show well-known problems that are not specific to neurointensive medicine but to numerous residency programs: the increasing concentration of work with less time required for practical training at the patient's bedside [8]. A large proportion of working time is spent on documentation and non-medical tasks [12]. Knowledge is mainly acquired through self-study mostly in leisure time [8]. This is a general problem far beyond the national borders. Unfortunately, there are few similar studies that allow comparisons between different countries.

The list of neurointensive care wards in Germany is in line with the number conducted by Busse et al. [6]. The response rate of 59,8% was higher than expected considering the narrow inclusion criteria. Because a few larger clinics had multiple physicians participating, these clinics may be slightly overrepresented in the results. It is also possible that the participation rate was higher among motivated physicians (both because they are particularly satisfied or dissatisfied with their previous working conditions). Because recruitment in some cases depended on the assistance of the chief physicians, they had some influence on the selection of participants.

Based on our results, we can identify some key points that can be used to improve training in neurointensive care and guide curriculum development.

### Training period

A sufficiently long and sensibly structured training period is of great importance for successful education in intensive care medicine [13, 14]. In reality, unfortunately, this time usually lasts less than a week. Many hospitals use an internship in anesthesiology to practice techniques like anaesthesia and airway-management.

Many residents asked for a standardized, written training concept. Answers of instructors and residents diverged. This indicates, that training concepts as well as SOPs often exist but are not regularly used and practiced.

### Ongoing training

Despite the additional workload, the residents long for regular seminars on ICU content and are willing to prepare them individually. In the USA, regular participation in seminars is fixed in the Program Requirements of the United Council for Neurological Subspecialties (UCNS) [15]. In Germany, a duty of further training only exists after passing the specialist examination [16]. In order to promote teaching and research, participation in continuing education courses, seminars and congresses must also be supported for residents in general.

### Curriculum requirements

In the USA, various authorities have already called for an ICU curriculum with defined training goals. Such a curriculum could relieve the burden on the teachers, since they would not have to design. Simultaneously, educational standards would be defined [17]. Due to the variability of the residency programs, such a curriculum must define specific goals and allow interpretation at the same time [8].

### Feedback

Feedback on clinical performance is essential for the learning success of residents [18] and for creating an active learning environment [19]. It enhances the self-confidence of residents in difficult clinical situations and helps them to evaluate their training as being of higher quality [19–21].

Despite the high demand of the residents for further training interviews, the training logbook only requires one formal feedback interview per year during the five-year neurological residency. Regarding the short period of rotation to ICU, the feedback interview rarely takes place during that time. In the USA, at least during the two-year neurointensive fellowship, semi-annual feedback sessions are planned [15].

The regular receipt of feedback is generally positively evaluated by residents [21–23], but at the same time feedback is rarely actively requested [21]. Teachers also see feedback as an essential component of good continuing education, but often assess the need for it as less than it actually is. This is also indicated by the significant difference in the response behavior of our study participants regarding the questions of whether feedback is regularly given or mistakes are discussed. Furthermore, there is often a reluctance to express (constructive) criticism in order not to unsettle or offend employees. Feedback in a short and non-specific form should be preferred [21, 23]. It must be required for an integral part of clinical teaching.

### Simulation training

The benefit of simulation training as a teaching method for residents has already been proven in numerous studies [24, 25]. It helps to increase the safety and efficacy of procedures, e.g. resuscitation or thrombolysis in acute ischemic stroke [26, 27]. Additionally, not only theoretical knowledge but also conversation with patients, colleagues and staff can be increased [22, 28–30]. Participants in our study believe their participation in simulations are extremely effective and attribute a great influence on their further training.

To reducing anxiety, simulation training has also been used in other projects to improve the self-confidence of residents and to improve the work-life balance, job satisfaction and the working atmosphere [14, 27, 29]. These are extremely important aspects for improving work in neurointensive medicine. Interest and attraction of work leads to retaining employees in the long term.

Our data shows that the teaching method "simulation" is already established in some hospitals, but many residents are not able to participate during their rotation because it is performed too rarely. Furthermore, it can be assumed that no further training units exist beyond obligatory resuscitation training.

Neurointensive medicine is a field with deep roots in other areas of medicine, such as neurosurgery, anaesthesiology and internal medicine. Many of the participants are asking for interdisciplinary teaching and, if possible, interdisciplinary rounds and case discussions regularly. In the UCNS Fellowship Program, interdisciplinary work is also considered a component of the program [15].

### Internal evaluation of the programs

Many of the participating residents did not know whether their internal ICU training has ever been evaluated. The advantages of such an evaluation are obvious. It goes along with a higher satisfaction of the residents and a better educational atmosphere [14]. The professional societies in the USA and many European countries have already recognized this importance and demand a systematic quality assessment of the programs [7, 15]. As long as this has not yet been widely implemented by a higher authority, instructors and residents are called upon to commit themselves to in-house teaching evaluation.

### Examinations

The same applies to knowledge checks during the further training period, which is advocated in Europe by the European Union of Medical Specialists (UEMS), for example [7]. Such reviews have already been evaluated by residents in several studies as a positive learning-factor. Moreover, it is known that examinations have a positive influence on learning success ("assessment drives learning") [14, 31, 32]. At the same time, such examinations should also be taken with caution, since they can be accompanied by an increased workload, especially if they take place too frequently and too extensively [31].

### Requirements for the instructors

Nearly all of the instructors of our study stated that they enjoy working as clinical trainers—even if they did not consider the conditions for continuing education at their clinic to be optimal. Planning and implementation of ICU training is rarely given its own time frame. The teaching should even be planned in addition to their regular work by senior physicians and specialists. Training in didactics had only been given to less than one third of those surveyed. Many instructors would request for feedback on their own teaching methods, even if the feedback is given by other teachers. Feedback is also useful to assess the effectiveness of one's own methods and change them if necessary [19, 33].

For this reason, the UCNS Common Program Requirements state that clinical educators must be given "time and authority" for supervision and evaluation of teaching [15]. Similar requirements would be desirable in Germany, too.

### Change in requirements of physicians

The generation of young physicians demands a work-life balance, adequate teaching and supervision, and a positive feedback culture [34]. This was also reflected by the residents in the interviews.

The family planning of young physicians becomes more difficult as long as no part-time working models exist (or are known to the employees) or rotations are not determined at an early stage. This may explain the conspicuously small proportion of parents among residents in our study. The increasing number of female physicians and equal partnerships will force institutions to create modern working time models and to plan definite rotation plans [3].

Overall, mental health plays an increasing role in everyday work, especially in younger physicians [14, 35, 36]. The desire for mentoring programs should be taken seriously as this was frequently mentioned in our study. Likewise, the lack of time and feedback reported by many residents should be addressed in a solution-oriented manner. It has been shown, that these factors, together with a negative learning experience, are associated with a higher incidence of depressive symptoms [13, 37]. It is necessary to support the residents as a mentor, especially in critical situations (familiarization period, first duty, "tragic" patient cases).

### Concepts of the professional societies

In Germany, DGN and DGNI have taken up the topic of neurological ICU training. A logbook for intensive care rotation is currently being prepared and is expected to be published in 2022. The fact, that the capacity for the six-month intensive care rotation can rarely be guaranteed at most clinics must urgently be addressed. One solution proposed here would be a clinic-network to advertise vacancies for intensive care rotation. Further residents might carry out their rotation at another hospital without having to change their employer. In the USA, such “away electives” are already common in the neurointensive wards of other hospitals [8].

## Conclusion

The present study provides an overview of the current status of neurointensive medical training during the five-year residency. In many cases, personnel and time resources limit the feasibility of promising concepts. Despite these limitations, the motivation of trainers and the enthusiasm of the residents for intensive care medicine is very high. Not at least due to the current pandemic, continuing education in neurointensive medical topics is increasingly important and must therefore be urgently promoted.

**Table:** 

Based on our findings, recommendations for action were formulated that could serve as guidelines for further development:
- Establish an orientation period of at least two weeks; during this period, the resident should be additionally scheduled and accompanied by a specialist
- Development of a training checklist: Determine which procedures, machines and treatment occasions should be mastered after the orientation period, possibly knowledge check at the end of the orientation period
- Promote continuing education opportunities: establish regular in-clinic or inpatient continuing education courses; financially promote participation in external courses and congresses and, if necessary, allow time off for these
- Simultaneously with the induction checklist, create a curriculum that defines what skills are to be acquired during the ICU rotation; regular review by instructor and resident to determine if these goals are being met
- Creating a feedback culture in which constructive feedback by specialists and among peers contributes to individual and team development; at least two feedback interviews in a protected setting during the rotation
- Train difficult interventions/situations on the model or in simulation: airway protection, resuscitation, conversation techniques, etc. as part of the curriculum
-Improve communication: create a platform for information about existing teaching opportunities and residency information
- Evaluate the residents’ learning success e.g. by short informal knowledge checks as well as evaluation by the residents at the end of the rotation
- Teach the trainer: enable your medical staff to receive didactical training and give them time to learn

## Data Availability

The datasets used and/or analysed during the current study are available from the corresponding author (Ann-Kathrin Ernst) on reasonable request.

## Abbreviations

NICM: Neurological intensive care medicine
ICU: Intensive care unit
NICU: Neurological intensive care unit
EAN: European Academy of Neurology
DGNI: Deutsche Gesellschaft für Neurointensiv- und Notfallmedizin (German Society for Neurointensive and Emergency Medicine)
DGN: Deutsche Gesellschaft für Neurologie (German Society for Neurology)

## References

[1] Foerch C, Misselwitz B, Sitzer M, Steinmetz H, Neumann-Haefelin T (2008). The projected burden of stroke in the German federal state of Hesse up to the year 2050. Dtsch Arztebl.

[2] Vonck K, Garrez I, De Herdt V, Hemelsoet D, Laureys G, Raedt R (2020). Neurological manifestations and neuro-invasive mechanisms of the severe acute respiratory syndrome coronavirus type 2. Eur J Neurol.

[3] Bundesärztekammer. Figures and tables on the physician statistics of Figures and tables on the physician statistics of the German Medical Association as of 31.12.2019 [in German]; 2019 [cited 19 Nov 2020]. Available from: URL: https://www.bundesaerztekammer.de/ueber-uns/aerztestatistik/aerztestatistik-2019/.

[4] Deuschl G, Müllges W. Statement intensive care medicine [in German]; 2020.000Z [cited 8 June 2020]. Available from: URL: https://www.dgn.org/rubrik-dgn/stellungnahmen-der-dgn/173-stellungnahme-intensivmedizin.

[5] Bundesärztekammer. (Model) Advanced Training Regulations 2018 [in German]; 2020 [cited 19 Nov 2020]. Available from: URL: https://www.bundesaerztekammer.de/fileadmin/user_upload/downloads/pdf-Ordner/Weiterbildung/20200428-MWBO-2018.pdf.

[6] Busse O, Hillmann S, Grond M, Neurointensivmedizin in Deutschland (2018). Ergebnisse einer Begehung von Intensivstationen [Neurointensive care in Germany. Results of an inspection of intensive care units]. Nervenarzt.

[7] Kleineberg NN, van der Meulen M, Franke C, Klingelhoefer L, Sauerbier A, Di Liberto G (2020). Differences in neurology residency training programmes across Europe – a survey among the residents and research fellow section of the european academy of neurology national representatives. Eur J Neurol.

[8] Sheth KN, Drogan O, Manno E, Geocadin RG, Ziai W (2012). Neurocritical care education during neurology residency: AAN survey of US program directors. Neurology.

[9] Dhar R, Rajajee V, Finley CA, Maas MB, James ML, Kumar AB (2017). The state of neurocritical care fellowship training and attitudes toward accreditation and certification: a survey of neurocritical care fellowship program directors. Front Neurol.

[10] Deutsche Gesellschaft für Neurointensiv- und -Notfallmedizin. Verzeichnis Neurointensivstationen [cited 13 Sep 2019]. Available from: URL: https://www.dgni.de/verzeichnis-neurointensivstationen.html.

[11] Mayring, P. Qualitative content analysis: theoretical foundation, basic procedures and software solution. Klagenfurt, 2014. http://nbn-resolving.de/urn:nbn:de:0168-ssoar-395173.

[12] Biesalski AS, Franke C, Sturm D, Behncke J, Schreckenbach T, Knauß S et al. Germany-wide evaluation of continuing medical education in clinical neurology [in German]. Nervenarzt. 2018;89(12):1378–87. Available from: URL: https://www.bundesaerztekammer.de/fileadmin/user_upload/downloads/pdf-Ordner/Statistik2019/Stat19AbbTab.pdf. 10.1007/s00115-018-0547-8 29872878

[13] Pereira-Lima K, Gupta RR, Guille C, Sen S (2019). Residency program factors associated with depressive symptoms in internal medicine interns: a prospective cohort study. Acad Med.

[14] Lee N, Appelbaum N, Amendola M, Dodson K, Kaplan B. Improving resident well-being and clinical learning environment through academic initiatives. J Surg Res 2017 [cited 11 Jul 2020]; (215):6–11. 10.1016/j.jss.2017.02.054 28688662

[15] United council for neurologic subspecialties. UCNS Common Program Requirements. Available from: https://www.ucns.org/Online/Accreditation/Program_Requirements.aspx.

[16] Bundesärztekammer. Model further training regulations [in German]; 2020.000Z [cited 1 Aug 2020]. Available from: URL: https://www.bundesaerztekammer.de/fileadmin/user_upload/downloads/_Muster-_Fortbildungsordnung_29052013.pdf.

[17] Mainous AG, Fang B, Peterson LE (2017). Competency assessment in family medicine residency: observations, knowledge-based examinations, and advancement. J Grad Med Educ.

[18] Clynes MP, Raftery SEC. Feedback: An essential element of student learning in clinical practice 2008 [cited 11 Jul 2020]. Available from: URL: https://reader.elsevier.com/reader/sd/pii/S1471595308000206?token=4F0C1824A72E858CB29197FDFFBAC34DEBB91324D56215EDA95D82672D39531A461FBECC18F86632E9A349D4AC078F24. 10.1016/j.nepr.2008.02.003 18372216

[19] Sultan AS, Mateen Khan MA (2017). Feedback in a clinical setting: A way forward to enhance student's learning through constructive feedback. J Pak Med Assoc.

[20] Hattie J, Timperley H (2007). The Power of Feedback. Rev Educ Res.

[21] Alves de Lima AE (2008). Constructive Feedback. A Strategy to Enhance Learning Medicina (B Aires).

[22] Collins K, Hopkins A, Shilkofski NA, Levine RB, Hernandez RG. Difficult Patient Encounters: Assessing Pediatric Residents' Communication Skills Training Needs. Cureus. 2018;10(9):e3340. 10.7759/cureus.3340 PMC6248659 30473973

[23] Wolpaw J, Saddawi-Konefka D, Dwivedi P, Toy S (2019). Faculty underestimate resident desire for constructive feedback and overestimate retaliation. J Educ Perioper Med.

[24] Hocker SE, Wijdicks EFMa. Simulation in Acute Neurology. Amsterdam: Elsevier. 2018.

[25] Low XM, Horrigan D, Brewster DJ (2018). The effects of team-training in intensive care medicine: A narrative review. J Crit Care.

[26] Boyle WA, Murray DJ, Beyatte MB, Knittel JG, Kerby PW, Woodhouse J (2018). Simulation-based assessment of critical care "front-line" providers. Crit Care Med.

[27] Tahtali D, Bohmann F, Rostek P, Misselwitz B, Reihs A, Heringer F (2016). Crew-ressource-management und simulatortraining in der akuten Schlaganfalltherapie. Nervenarzt.

[28] Siddharthan T, Soares S, Wang HH, Holt SR (2017). Objective structured clinical examination-based teaching of the musculoskeletal examination. South Med J.

[29] Braksick SA, Kashani K, Hocker S. Neurology Education for Critical Care Fellows Using High-Fidelity Simulation. Neurocrit Care. 2017;26(1):96–102. Available from: URL: https://link.springer.com/article/10.1007%2Fs12028-016-0293-3. 10.1007/s12028-016-0293-3 27389006

[30] Reed S, Kassis K, Nagel R, Verbeck N, Mahan JD, Shell R (2015). Breaking bad news is a teachable skill in pediatric residents: a feasibility study of an educational intervention. Patient Educ Couns.

[31] Finis D, Bramann E, Geerling G. Evaluation of a New Residency Program at the Department of Ophthalmology of the Heinrich Heine University Düsseldorf [in German]. Ophthalmologe. 2015;112(6):504–11. 10.1007/s00347-015-0078-7 26065527

[32] Wass V, van der Vleuten C, Shatzer J, Jones R (2001). Assessment of clinical competence. Lancet.

[33] Garcia I, James RW, Bischof P, Baroffio A (2017). Self-observation and peer feedback as a faculty development approach for problem-based learning tutors: a program evaluation - pubmed. Teach Learn Med.

[34] Roeth AA, Mille M (2018). Was wollen die jungen Chirurgen? moderne anforderungen an chirurgische chefs [what do young surgeons want? modern requirements for senior surgeons]. Zentralbl Chir.

[35] Beschoner P, Limbrecht-Ecklundt K, Jerg-Bretzke L (2019). Mental health of physicians [in German]. Nervenarzt.

[36] Jordan JT, Mayans D, Schneider L, Adams N, Khawaja AM, Engstrom J (2016). Education research: neurology resident education: Trending skills, confidence, and professional preparation: trending skills, confidence, and professional preparation. Neurology.

[37] Ogawa R, Seo E, Maeno T, Ito M, Sanuki M, Maeno T. The relationship between long working hours and depression among first-year residents in Japan; 2018 [cited 2021 Jul 11.921Z]. Available from: URL: https://www.ncbi.nlm.nih.gov/pmc/articles/PMC5870810/. 10.1186/s12909-018-1171-9 PMC5870810 29587738

